# CerealsDB—new tools for the analysis of the wheat genome: update 2020

**DOI:** 10.1093/database/baaa060

**Published:** 2020-08-04

**Authors:** Paul A Wilkinson, Alexandra M Allen, Simon Tyrrell, Luzie U Wingen, Xingdong Bian, Mark O Winfield, Amanda Burridge, Daniel S Shaw, Jan Zaucha, Simon Griffiths, Robert P Davey, Keith J Edwards, Gary L A Barker

**Affiliations:** 1 School of Biological Sciences, University of Bristol, Bristol Life Sciences Building, 24 Tyndall Avenue, Bristol, BS8 1TQ, UK; 2 Earlham Institute, Norwich Research Park, Norwich NR4 7UZ, UK; 3 John Innes Centre, Norwich Research Park, Norwich NR4 7UH, UK; 4 Department of Bioinformatics, Wissenschaftszentrum Weihenstephan, Technical University of Munich, 85354 Freising, Germany; 5 Institute of Systems, Molecular and Integrative Biology, Faculty of Health and Life Sciences, University of Liverpool, BioSciences Building, Crown Street, Liverpool, L69 7ZB, UK

## Abstract

CerealsDB (www.cerealsdb.uk.net) is an online repository of mainly hexaploid wheat (*Triticum aestivum*) single nucleotide polymorphisms (SNPs) and genotyping data. The CerealsDB website has been designed to enable wheat breeders and scientists to select the appropriate markers for research breeding tasks, such as marker-assisted selection. We report a large update of genotyping information for over 6000 wheat accessions and describe new webtools for exploring and visualizing the data. We also describe a new database of quantitative trait loci that links phenotypic traits to CerealsDB SNP markers and allelic scores for each of those markers. CerealsDB is an open-access website that hosts information on wheat SNPs considered useful for both plant breeders and research scientists. The latest CerealsDB database is available at https://www.cerealsdb.uk.net/cerealgenomics/CerealsDB/indexNEW.php.

## Introduction

The rapid expansion in human population size has driven food security up the global political agenda, making it one of the primary issues that governments are keen to address. Bread or hexaploid wheat (*T. aestivum*) is a cereal crop grown worldwide and is a major source of nutrition for both humans and livestock. Developing novel varieties of wheat that result in increased yield combined with other important traits such as disease and drought resistance that could potentially contribute to its long-term sustainability is necessary to guarantee the continued supply of this economically important crop.

Single nucleotide polymorphisms (SNPs) are defined as single base pair (bp) changes in a DNA sequence and are the most common form of sequence variation between individuals within a species. As a result of their high frequency in the wheat genome, SNPs have been used to develop markers such as KASP assays or array probes for use in marker-assisted selection of important agronomic traits that breeders are attempting to introgress into future elite wheat cultivars (1).

CerealsDB initially hosted a dataset of wheat expressed sequence tag sequences and evolved to include a database of Diversity Arrays Technology markers, validated SNPs and links to a draft genome of the wheat variety ‘Chinese Spring’. The second iteration of the site aimed to provide a more intuitive browsing experience and displayed the underlying data in a format that was easy to understand and could be manipulated by non-expert users (2).

The third version of CerealsDB was released in 2016, following an extensive redesign to accommodate new SNP datasets validated on a range of genotyping platforms. These included KASP and array-based platforms (3, 4) and more recently included SNPs used in a targeted genotyping by sequencing method (5). The website currently receives in excess of 60 000 unique visits per month and has a global audience (Supplementary Figure 1). This version of the site also contained links to a functional SNP database and webtools for visualizing genotyping data. In addition, web services were developed to expose the underlying data in the CerealsDB database to facilitate integration with other online genomic data resources, making use of the Grassroots application programming interface (API) developed by the Earlham Institute, which is interoperable and conforms to a set of common data standards (6). In order to improve the functionality of the website and respond to the requirements of users, we have developed several tools that are expected to be useful to the wheat genomics and plant breeding communities.

Here, we present the latest features and data updates to the CerealsDB site, including webtools that allow users to search for information on wheat accessions, tools that cross-reference SNPs that have been validated on multiple genotyping platforms and a new database that allows users to search for pre-identified quantitative trait loci (QTL), which link the genotypic information to the phenotypic data. We also present online phylogenetic trees, based on Axiom® array genotype scores, which allow an exploration of the genetic relationships of the scored accessions, and tools to explore putative introgressions between elite wheat varieties and wheat relative and progenitor species.

## Data upgrade

### Data summary and statistics

#### SNP statistics

SNPs from the Axiom® genotyping platform are currently the most abundant category of SNPs in CerealsDB with 819 571 held in the database. These SNPs are located on 91 848 wheat genome contigs that were assembled from 454 sequence data from normalized and non-normalized cDNA libraries prepared from RNA from wheat variety ‘Chinese Spring’ line 42 (7).

The average number of SNPs per contig was 6, with a maximum number of 157 in a single contig. The number of SNP-containing contigs, the average number of SNPs per contig and the minimum and maximum number of SNPs found within contigs for each wheat chromosome are displayed in Supplementary Table 1.

Chromosome 2D contained the greatest number of contigs (8216) that contained SNPs and chromosome 6B contained the least number of contigs (4775).

#### Annotation of Axiom® SNPs

Axiom® SNPs have been derived from wheat exonic sequences and are therefore strongly associated with the gene content in the wheat genome. We have improved the utility of these SNPs by annotating their surrounding sequence by homology searches against common protein databases using BLASTX (8).

The number of SNP sequences that could be annotated with a top BLASTX hit with Embryophyta protein database was 513 847 (62.7%) and the number that returned a ‘No hit’ was 305 725 (37.3%). There were 139 791 (17.1%) of these SNP sequences that produced an alignment to a ‘hypothetical protein’, and 98 778 (12.1%) to ‘predicted protein’, making these the most common annotations.

The contig sequences in which each SNP is located (79 509 contigs in total) were aligned to the NCBI non-redundant (nr) database using default BLASTN parameters and the top hits were parsed. The putative protein sequences derived from the top BLAST hits were parsed for their GenBank accession codes, which were then used to query the UniProt REST API using custom Perl scripts to enable programmatic access to Gene Ontology (GO) information. A custom Perl script was written to automate the submission of accession codes to the QuickGO REST web services (9) provided by the European Bioinformatics Institute (https://www.ebi.ac.uk/QuickGO/api/index.html). This script parses the UniProt protein accession and searches QuickGO. The GO results were then parsed to extract the GO accession.

A new MySQL table was created in CerealsDB to store this information, including fields for BLAST accession, contig ID in which the SNP is located, BLAST annotation, UniProt ID and GO (if available). There were 274 569 (33.5%) SNP contigs that could not be annotated as they did not produce an alignment to the nr database. Of these remaining contigs, a significant proportion (171 392 or 20.9%) were categorized as coding for hypothetical proteins.

Genes encoding disease resistance proteins were well represented with 10 619 sequences. Other common annotations of gene functions included subtilisin-like proteases (1058), secologanin synthases (905), pentatricopeptide-repeat-containing proteins (951), SRG1 proteins (717), peptide transport proteins (703), peroxidase (572), receptor protein kinases (547) and MYB-related proteins (495) and cytochrome p450 proteins (3681). The top 25 represented proteins are displayed in Supplementary Table 2.

CerealsDB SNPs have also been annotated with FATHMM scores that can highlight the functional consequences of non-coding and coding single nucleotide variants. FATHMM scores predict the magnitude of effect of SNPs causing single amino acid variants (SAVs, also known as missense variants) and their probability of affecting protein function and thus phenotype. SNPs that have a FATHMM score less than −3 are considered to have a deleterious effect on protein function and are highlighted in red (the threshold was chosen based on human SAVs, for which more manual annotations are available). The procedure for assigning FATHMM scores to SAVs was the following: if the contig the SNP belonged to was at least 15 amino acids in length, this allowed building a reliable hidden Markov model (HMM) for the sequence; for this purpose, the JackHMMER program from the HMMER 3 package (10) was run (for two iterations; -N 2 flag) to search for homologous sequences within the UniRef50 database (11) and create profile HMMs from the multiple sequence alignments. For each SAV, the probabilities of the wild-type and mutant amino acids were extracted from the Dirichlet mixtures (12) describing the profile HMMs and used to calculate the unweighted FATHMM score as defined in Shihab *et al.* (13). A search page has been created where users can query the functional SNP data by SNP name, by a generic search term or by FATHMM score and is located at the following URL: https://www.cerealsdb.uk.net/cerealgenomics/CerealsDB/funcSNPs_select.php.

#### Genotyping data

CerealsDB hosts a range of genotyping datasets that were generated using different genotyping platforms. Many of these genotyping datasets are from mapping populations that consist of 3973 genotyped individuals across 25 mapping populations. The largest of these is the Axiom® 820K HD array SNPs with 476 varieties, and 458 individuals from two mapping populations (AvalonxCadenza and ChineseSpringxParagon) are currently genotyped. The Axiom® 35K breeders array has 1962 varieties genotyped along with 460 individuals from 23 mapping populations. There are also genotyping data for the 36 711 SNPs included on the Axiom® Wheat-Relative Genotyping array (14), based on data from screening 10 wild relative species (*Aegilops mutica*, *A. speltoides*, *A. tauschii*, *T. timopheevii*, *T. urartu*, *Secale cereale*, *Thinopyrum bessarabicum*, *T. elongatum*, *T. intermedium* and *T. ponticum*). The wheat-relative array was used to produce genotyping results for 278 wheat relative and progenitor species, which are held in CerealsDB. The total number of accessions genotyped for each of the Axiom® arrays and mapping populations is displayed in Table 1. The genotyping data can be accessed from the following URL: (https://www.cerealsdb.uk.net/cerealgenomics/CerealsDB/array_info.php).

**Table 1.** List of collections and mapping populations that have been genotyped on the Axiom® arrays

Category	Number of varieties/individuals
820K_array	476
35K_breeders_array	1962
35K_relatives_array	278
AvalonxCadenza	185
ParagonxTetraploid BC1F5	189
Doonan_sphaerococcumxCapelle	75
Boden_tilling_samples	141
ChineseSpringxParagon	273
NIAB_INEW_and_ERYCC	235
NIAB_smel	92
OakleyxGatsby	74
ParagonxStarkeshortday	136
ParagonxWatkinsINEW	458
ParagonxBaj	169
ParagonxBecard_kachu	97
ParagonxCimcog47	409
ParagonxCimcog49	369
ParagonxMisr1	95
ParagonxPfau	97
ParagonxSuper152	97
ParagonxWaxwing	95
ParagonxWyalkatchem	88
ShamrockxShango	90
WeebillxCimcog03	133
WeebillxCimcog32	90
WeebillxCimcog56	95
Yumai34xClaire	100
Yumai34xUkrainka	91
Total	6689

A large proportion of the CerealsDB genotyping datasets were produced for 25 bi-parental mapping populations consisting of 3973 genotyped individuals across them. These populations are described in more detail in Table 2.

**Table 2.** Information on mapping populations genotyped for CerealsDB

Mapping population	Comment
Avalon x Cadenza	This is a population of doubled-haploid individuals produced within WGIN (www.wgin.org.uk) as a UK reference population. It was derived from F1 progeny of a cross between the cultivars Avalon and Cadenza. The parents were originally chosen to contrast for canopy architecture traits.
ChineseSpringxParagon	This population was developed as SSD to generation F6 produced within WGIN (www.wgin.org.uk). The parents are CS and Par. CS is the international reference genome and Par is the common parent in many UK efforts; both are sequenced. Par is a modern UK spring wheat and is used as the common parent of the landrace bread wheat nested association panel (15), with CSxPar being part of.
ParagonxStarke Short Day	This population was developed as SSD to generation F4 with Par and Starke parents, produced within DFW (www.designing-future-wheat.ac.uk). Starke is a Swedish variety released between 1960 and 1970, which exhibits a stronger than usual delay in heading time when grown under short days. Starke is in the Gediflux Northern European Wheat collection.
Paragonxtetraploid BC1F5	This population was created with Par and tetraploid (*durum* and *dicoccoides* as donor parents), as part of the DFW synthetics pillar. Seeds for this population have been deposited at the GRU.
Yumai 34 x Claire	This population was developed as SSD to generation F5 with Yumai 34 and Claire parents. Yumai 34 is a Chinese cultivar shown to contain high-soluble fiber in the HEALTHGRAIN project, Claire is a very successful UK biscuit wheat. Seeds for this population are available from the GRU.
Yumai32 x Ukrainka	This population was developed as SSD to generation F5 with Yumai 34 and Ukrainka parents. Yumai 34 is a Chinese cultivar shown to contain high-soluble fiber in the HEALTHGRAIN project; Ukrainka is a Hungarian variety that is productive and adaptable, with excellent baking quality. (Shewry, 2009). Seeds for this population are available from the GRU.
Paragon x Baj	This population was developed as SSD to generation F4 with Par and Baj parents as part of the IWYP (https://iwyp.org). Baj is a CIMMYT line (GID 5106304), very early maturing, heat- and drought-tolerant and well adapted to eastern Gangetic plains.
ParagonxBecard/Kachu	This population was developed as SSD to generation F5 with Par and Becard/Kachu parents as part of IWYP. Becard/Kachu is a CIMMYT line (GID 6174886) that performed well in trials in Obregon under the wheat yield consortium led by Matthew Reynolds. Kachu, another CIMMYT line for cultivation in northwestern India, is derived from Bacanora. Becard is derived from Weebill.
Paragon x CIMCOG47	This population was developed as SSD to generation F5 with Par and CIMCOG 47 parents as part of IWYP. CIMCOG 47 is CIMMYT line (GID 6000921).
**Paragon x CIMCOG49**	This population was developed as SSD to generation F5 with Par and CIMCOG 49 parents as part of IWYP. CIMCOG 49 is CIMMYT line (GID 6175024).
Paragon x MISR1	This population was developed as SSD to generation F5 with Par and MISR1 parents as part of IWYP. MISR1 was released by CIMMYT (GID 4902859) for Egypt, Afghanistan and Pakistan and carried Sr25 segment.
Paragon x Pfau	This population was developed as SSD to generation F5 with Par and Pfau parents as part of IWYP. Pfau is an early maturing CIMMYT variety (GID 12989).
Paragon x Super 152	This population was developed as SSD to generation F5 with Par and Super 152 parents as part of IWYP. Early maturing variety that performed very well in CIMMYT ESWYT trials, identified as having potential for wide adaptation in India (GID 5390612).
Paragon x Synth Type	This population was developed as SSD to generation F5 with Par and a CIMMYT synth type (GID 6176523) parents as part of IWYP.
Paragon x Waxwing	This population was developed as SSD to generation F5 with Par and Waxwing parents as part of IWYP. Waxwing is a CIMMYT line (GID 334864).
Paragon x Wyalkatchem	This population was developed as SSD to generation F6 with Par and Wyalkatchem parents as part of IWYP. Wyalkatchem is an Australian variety with CIMMYT parents (GID 3828890) very widely grown until recently.
Weebill x CIMCOG03	This population was developed as SSD to generation F5 with Weebill1 and CIMCOG 03 parents as part of IWYP. CIMCOG 03 is a CIMMYT line (GID 6175208).
Weebill x CIMCOG32	This population was developed as SSD to generation F5 with Weebill1 and CIMCOG 32 parents as part of IWYP. CIMCOG32 is a CIMMYT line (GID 4774392).
Weebill x CIMCOG56	This population was developed as SSD to generation F5 with Weebill1 and CIMCOG 56 parents as part of IWYP. CIMCOG 56 is a CIMMYT line (GID 6179253).

SSD indicates single seed descent; CS, Chinese Spring; Par, Paragon.

Genotyping data have been converted into variant call format (VCF) files for the 820K array, 35K wheat breeders and 35K wheat-relative arrays and are hosted on CerealsDB.

#### Varietal information

CerealsDB now includes a varietal database providing descriptions on all wheat varieties and related species currently genotyped on the 820K HD array and 35K wheat-relative and breeders’ arrays. These data are especially useful to wheat breeders and consist of information on alternate names for the varieties, species ploidy, country of origin and the source of the material. The varietal database also includes information on the sample type, for example categorization of the variety as a ‘cultivar’—a wheat variety that has been generated via a breeding programme—in contrast to a ‘landrace’ that is locally adapted germplasm that has not been systematically bred. The ‘sample type’ field in the database includes information on specific wheat research or breeding programmes where applicable.

According to the Axiom® genotyping array used, genotype data are stored in the ‘820K array’ or the ‘35K array’ tables in the database. Each table contains 12 columns and there are currently 975 varieties in the 820K array table, compared with 6144 varieties in the 35K array table. The varieties come from a total of 51 or 57 countries, respectively, with UK varieties being the most highly represented in both tables (512 in the 820K array table and 1971 in the 35K array table). The distribution of varieties by country is displayed in Supplementary Figure 2.

The 820K array data come from 855 hexaploid wheat varieties, 65 tetraploid wheat and wheat relatives, 47 diploid species and a single decaploid species (*T. ponticum*). The majority of the 35K array genotyping data are derived from hexaploid wheat varieties (6080); however, there are also data for 37 tetraploid species and 11 diploid species. We were unable to assign a ploidy level to some species (5 on the 820K array and 16 on the 35K array) and have categorized them as ‘unknown’. There are 820K array data for 86 wheat relative and progenitor species (mainly *Aegilops*, *Thinopyrum* and related *Triticae*) compared with the 35K array that has data for only 11 wheat relatives, all of which are varieties of *A. tauschii*.

#### QTL data

As part of Wheat Improvement Strategic Programme and designing future wheat (DFW) programs, field trials were conducted on a set of bi-parental populations (Table 3). The segregating populations are part of the wheat landrace NAM population described in Wingen *et al.* (15). Population development, KASP genotyping and genetic mapping were conducted as described, with the exception of population Par x Watkins 1 190 094 (PW094), which was genotyped using the Breeders’ 35K Axiom® array (16). Due to the large size of the dataset, genetic mapping was conducted with MSTmap Online (17) (http://www.mstmap.org/) using an LOD 10 threshold and manually separating the linkage groups as markers carry information to which chromosome they belong.

**Table 3.** List of bi-parental populations and their characteristics

Population name	Number of RILs	Linked marker loci in genetic map	Genotype
ParW094	109	3088	Axiom®
ParW103	94	172	KASP
ParW308	80	281	KASP
ParW471	92	281	KASP
ParW670	94	185	KASP
ParW740	94	164	KASP
ParW749	94	221	KASP

All populations share ‘Paragon’ as common parent and have a Watkins stabilized bread wheat landrace accession (http://gru-lims-test.nbi.ac.uk/search-browseaccessions.php?idCollection=39) as second parent.

Populations were developed as SSD up to generation F4 as described in (15) and genotyped with KASP markers apart from one exception.

W<number> indicates Watkins accession 1190<number>.

Populations were grown as part of the DFW WP3 multiplication plots in winter sown un-replicated field trials in the 2016/2017 season in 1 m^2^ plots with 20% controls at the John Innes Farm at Bawburgh, Norwich, UK (52.63 degree N, 1.18 degree E), without nitrogen treatment. The populations were phenotyped for plant height (PH) in centimeter as measured from the soil surface to the highest point of the plant and for heading date (HD) in days after 1 May, when in half of the plot, half of the ears had emerged from the flag leaf. QTL mapping was performed for trait data and genotype and map information, using the package QTL (18) (version 1.42.8) from the R software suite (vs. 3.5.2) (R core team 2015) taking the cross-type and generation number of the populations into account. QTL analyses were performed in two steps: putative QTL were identified in an initial single QTL scan and subsequently tested in a final multiple QTL model using a significant threshold calculated from the data distribution.

Genotype and phenotype data are available from http://wisplandracepillar.jic.ac.uk/results_resources.htm, and seed for the populations is available by request.

The QTL database currently contains 65 QTL for 9 wheat phenotypes, consisting of 13 QTL for PH, 7 QTL for heading time, 8 QTL for grain length, 13 QTL for grain section area, 7 QTL for grain width, 5 QTL for grain yield, 1 QTL for HD, 1 QTL for presence of awns and 10 QTL for thousand grain weight.

For each QTL, markers identified at the start and end of the QTL confidence interval and at the QTL peak are given. Crop ontology (19) identifiers were assigned to help further classify data based on agriculture-related information and terminology related to phenotype, breeding, germplasm, pedigree and traits (http://www.cropontology.org/). Two web services were developed at the Earlham Institute to allow access to information on how the genotyping data relate to given markers. Both are part of the Grassroots infrastructure and hence use the Grassroots API and libraries for their operation. The first service is used to import and store the spreadsheets containing the genotyping information into a MongoDB database to store the relationships between the markers, the genotypes and the cross-parental data. The second service accepts queries for searching these data using the marker and/or population names. Clicking on the markers accesses the genotyping data by calling this web service (Supplementary Figure 3).

#### Phylogenetic trees

For the 820K array genotyping dataset, there were 773 301 (94.35%) SNPs that could be accurately assigned to a location on the International Wheat Genome Sequencing Consortium (IWGSC) RefSeq v1.0 whole genome assembly, compared with 31 926 (90.86%) for the 35K array.

A VCF file was generated for SNPs in accordance with version 4.1 specification with allelic information included. Genotypes were encoded with the Chinese Spring reference allele assigned to 0 and the alternative allele assigned to 1. There were 475 wheat varieties and wheat relative/progenitor species for the 820K genotyping dataset and 1962 wheat varieties that had been genotyped using the 35K array.

The VCF file was then submitted to the SNPhylo analysis pipeline, which excluded 664 280 (85.9%) SNPs due to their monomorphic nature, having a minor allele frequency less than 0.1 or a missing data rate greater than 0.1 (missing data are where a specific variety has failed to genotype IE encoded as −/− in the VCF file) compared with the 35K array genotyping data where 12 739 (39.9%) SNPs were excluded. The number of SNPs for each chromosome for the 820K array data is displayed in Supplementary Table 3 and for the 35K array data in Supplementary Table 4. A total of 58 553 SNPs was used in the 820K analysis and 15 627 in the 35K analysis.

The Newick strings generated by SNPhylo were imported into the Phylocanvas (20) package (version1.1) that allows the phylogenetic trees to be displayed online. The Phylocanvas JavaScript code was embedded into CerealsDB webpages to allow users to interactively explore the dendrograms (Figure 3).

#### Introgression identification

The assignment of a physical map position to the SNP markers was achieved by BLAST searching the probe sequences to the IWGSC whole genome assembly v1.0 (https://wheat-urgi.versailles.inra.fr/Seq-Repository/Assemblies). The identification of putative introgressions was performed by comparing the genotype calls for each of the 339 hexaploid lines with those of the 116 wheat relatives and progenitors over a 10 SNP window and calculating a percentage match. Analysis of control introgression lines indicated that a relative match of 0.4 or higher was indicative of an introgression in a hexaploid background. This threshold was chosen based on the screening of known introgressions such as 1B/1RS.

The next part of the introgression prediction pipeline takes the predicted scores for each chromosome from the previous step and uses a sliding window of 10 SNPs along the chromosome and calculates the average similarity scores for consecutive 10 SNP windows for each of the varieties, to identify putative regions of introgression along each chromosome. A summary file was then generated for each variety to identify regions of introgression for each chromosome. The summary file reports the chromosomal location and size of putative introgressions above 0.4 for each wheat-relative comparison. The analysis pipeline achieved this by looking for consecutive SNPs that had a relative match of 0.4 or higher and if the next SNP along did not have a relative match of 0.4 or greater, then the preceding SNP was classified as a small introgression. If successive SNPs all had a relative match of 0.4 or greater, then the distance between the first and last SNP with a match ≥0.4 was calculated to identify the approximate size of the introgression. For example, if five consecutive SNPs have a similarity score greater than 0.4, then the distance between the first and the fifth SNPs would be used to calculate the size of the introgression. In this way, it was possible to identify potential introgressions/deletions along each chromosome for every combination of hexaploid wheat variety and wheat relative/progenitor species.

#### CNV analysis

Copy number variation (CNV) analysis was performed using the Affymetrix CNV Tool software. CEL files from the Axiom® Wheat HD Genotyping Array were processed using Axiom® Analysis Suite as described above. The annotation file was generated using the Affymetrix Annotation Converter. CNV analyses were visualized in BioDiscovery Nexus Copy Number (El Segundo, CA, USA).

Introgression and CNV data can be visualized using the ‘Introgression Plotter’ tools located in the right-hand menu on the axiom genotyping page.

### Webtools

An explanation of updated webtools and their current URLs are displayed in Table 4. These webtools can also be accessed from the right-hand menu of the CerealsDB home page. The webtool page contains brief explanations of each webtool: https://www.cerealsdb.uk.net/cerealgenomics/CerealsDB/webtools.php

**Table 4.** New webtools and their locations on CerealsDB

Webtool function	URL
Retrieving genotyping data	http://www.cerealsdb.uk.net/cerealgenomics/CerealsDB/extract_genotypes.php
Searching varietal database	http://www.cerealsdb.uk.net/cerealgenomics/CerealsDB/select_varietal_info_SeedStor.php
Searching the QTL database	http://www.cerealsdb.uk.net/cerealgenomics/CerealsDB/select_QTL.php
Retrieving SNPs that align to related species	https://www.cerealsdb.uk.net/cerealgenomics/CerealsDB/FORM_SNPs_vs_related.php
Selecting Axiom® SNPs between two known locations	https://www.cerealsdb.uk.net/cerealgenomics/CerealsDB/axiom_extractor.php
Online dendrograms for Axiom® 820K and 35K arrays	https://www.cerealsdb.uk.net/cerealgenomics/CerealsDB/820K_dendrogram.php https://www.cerealsdb.uk.net/cerealgenomics/CerealsDB/35K_dendrogram.php
Search for putative introgressions/deletions	https://www.cerealsdb.uk.net/cerealgenomics/CerealsDB/search_introgressions.php

#### Retrieving genotyping data

While the previous iteration of CerealsDB included the genotyping data as downloadable Excel spreadsheets, the updated site now has a webtool that allows users to download genotyping information for a specific Axiom® SNP or for a specific wheat variety or wheat relative/progenitor species.

In the first step, the users must decide if they want to get genotype information for a variety or for a SNP. If the latter has been selected, users can search for allelic scores for any SNP on an Axiom® array by selecting one of the two arrays (either 820K or 35K) in the top selection box and providing the Axiom® SNP code (e.g. AX-94381147). Once the SNP code has been submitted, the webtool will list all varieties scored for the selected array with both the variety code generated by the Affymetrix array analysis pipeline and the genotype called. In addition, users can download the data as a tab-delimited text file.

The genotyping data can also be retrieved by searching for a specific variety in the first step mentioned above. This is accomplished by selecting an array or mapping population and then choosing a variety from a drop-down list; alternatively, users can enter a partial variety name and the field will autocomplete if a variety is present on that array (or mapping population). Users can then select genotyping results for probes on all chromosomes or a combination of chromosomes, and the webtool will display the genotype calls for the selected chromosomes, with the option of downloading the data in tab-delimited text format.

#### Searching varietal database

The varietal database can be searched via a simple webtool that allows users to select varieties genotyped on either the 820K or the 35K Axiom® array. Varieties can be selected from a drop-down list or the name can be typed into the entry field that auto-completes if the variety is present on the array. Alternatively, a general search term can be entered in this field, such as the country of origin or the source of the germplasm and the tool will retrieve varieties annotated with this information. For example, typing ‘Australia’ and searching the 820K array currently retrieves six varieties that were sourced from this country.

CerealsDB varieties have been linked to the Germplasm Resource Unit (GRU), a UK seed bank with a focus on crops, including wheat, brassica and peas. A recently developed API now connects CerealsDB to SeedStor, a database hosted at the GRU (21). This SeedStor API allows users to link any genotyped variety on CerealsDB with the correct GRU accessions providing a direct connection to the SeedStor website (https://www.seedstor.ac.uk/), which contains information like taxonomy, pedigrees, phenotypes and images if available and allows users to directly ordering seed for varieties of interest.

#### Searching the QTL database

**Figure 1.**
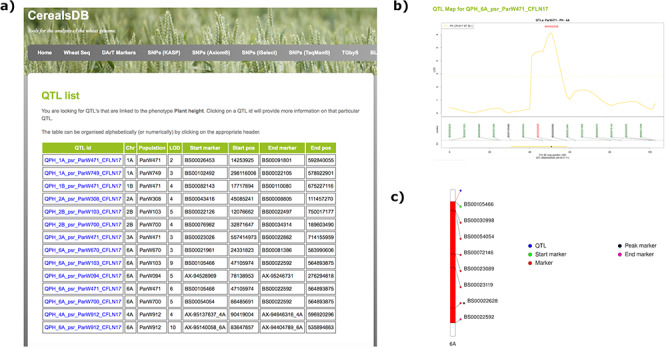
Searching the QTL database for a particular phenotype generates a table (a) displaying all QTLs for that phenotype along with chromosomal positions, population information and LOD scores. Clicking on an individual QTL links to QTL maps (b) where markers are shown at the start and end of the confidence interval of the QTL along with the peak marker. An ideogram of the chromosome (c) is also displayed where available showing the QTL highlighted in red and the chromosomal position of important markers are also highlighted.

The QTL database, currently holding QTL data identified in bi-parental populations of the DFW landrace NAM panel (wisplandracepillar.jic.ac.uk/results_resources.htm) can be searched online via a web interface. Currently, users can type in one of two phenotypes, ‘Plant height’ or ‘Heading time’, and information on all QTL present for either of these phenotypes is displayed in a table, including information on chromosome location, LOD score, QTL confidence interval start and end markers and the QTL peak marker, along with positions on the genetic map (Figure 1). Clicking on a QTL ID links to a page containing more information on that QTL, including physical positions of markers on the chromosome (based on alignment to the IWGSC assembly RefSeq v1.0) a genetic map of the markers associated with the QTL and links out to the region in EnsemblPlants (22) wheat browser. Ideograms of marker locations on wheat chromosomes were generated using the PhenoGram software (23). Crop ontology information is also provided with links out to the website http://www.cropontology.org/ that hosts agriculture-related information and terminology related to phenotype, breeding, germplasm, pedigree and traits (19).

**Figure 2.**
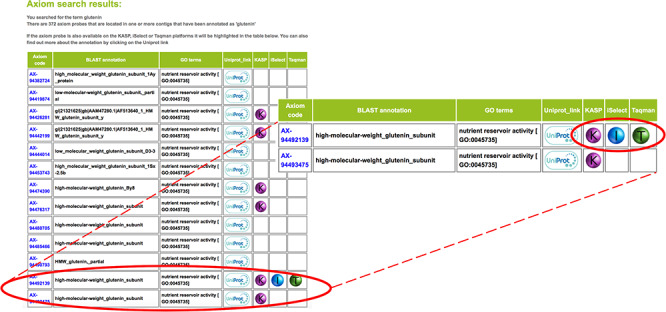
Colour-coded glyphs allow users to rapidly scroll through hundreds of results and identify cross-platform SNPs, which is a useful tool for plant breeders that often require ‘legacy’ markers for their breeding programmes.

The QTL database is predicted to be a useful tool for plant breeders looking for novel variation to cross into breeding programmes. For example, a breeder might be interested in the recently identified plant height (PH)-reducing locus *Rht24* on chromosome 6A (24, 25) as ‘*Rht24* appears in breeding programs from all countries of origin investigated, with increased frequency over the last decades, indicating that wheat breeders have actively selected for this locus.’ (25).

It seems reasonable to hypothesize that users can find different alleles for this locus in a QTL database as it has been speculated (24) that this locus is underlying a meta-QTL for PH on 6A in European elite germplasm (26). In the search for novel alleleic diversity, CerealsDB would be a good place to start, as it hosts information from geographically disparate accessions. In line with this, the QTL database holds QTL data from crosses between bread wheat landraces, from the diverse A E Watkins collection, and the European accession, ‘Paragon’. Searching the database with the key word ‘plant height’ currently brings up 13 PH QTL of which six are located on chromosome 6A. The position on the IWGSC RefSeq v1.0 is given for each QTL for the marker closest to the QTL peak and for the markers bordering the QTL confidence interval. Further markers within the confidence interval are listed and their position on the genome assembly can be retrieved by using the ‘Extract probes’ webtool. The alignment of the six PH QTL along the 6A chromosome and the comparison of their location to the position of the most significantly associated marker S3222505 (located at 420391134 Bp) and the recommended region for marker assisted breeding (25) reveals that the QTL confidence intervals overlap with that region or are near (Supplementary Figure 4). Given that local rearrangements in individual accessions in comparison with the reference sequence are frequent in wheat, QTL positions, which are genetically defined may appear at an unexpected distance to a candidate locus on the wheat reference genome. Therefore, all six QTL are potential candidates for alleleic variation of *Rht24*. Predicted alleleic effects are 2.6–5.2 centimorgans, with the increasing effect coming from the landrace parents. Depending on the combination with other *Rht* genes, and on the target environment of the breeding programme, these alleles could be useful to achieve an optimum PH. The usefulness of the QTL database will grow with the number of QTL for different populations, traits and environments deposited here.

#### Retrieving SNPs that align to related species

A webtool has been developed to find wheat SNP marker sequences on the genome sequences of related species. This webtool allows users to obtain the physical location of an SNP marker on the reference genomes of *Brachypodium*, *oryzae* (rice), *Sorghum* and of wheat (IWGSC RefSeq v1.0 whole genome assembly). This information can be useful for homology-based cloning approaches and rapid identification of candidate genes.

Users must enter an Axiom® SNP ID and can select a range in bps to retrieve all stored Axiom® marker SNPs within that range on either side of the query SNP. The Axiom® SNP marker sequences have been mapped to the reference genomes using BLASTN, and an e-value cut-off can be defined to exclude less reliable alignments. Users can choose a genome reference sequence and the SNP along with upstream and downstream flanking SNPs are displayed with positional information and the e-value score for that BLAST SNP alignment to the reference sequence. Other useful information includes the SNP status, i.e. is it a synonymous or non-synonymous SNP, and a FATHMM score (13) if this has been calculated.

If alternate SNP codes are present, these are also displayed, allowing users to quickly cross-reference for SNPs on other genotyping platforms. Clicking on the Axiom® code displays the sequence surrounding the SNP and links out to UniProt resources (27) if the sequence has been annotated.

#### Selecting Axiom® SNPs between two known locations

The ability to extract all stored SNPs that are located between two known SNPs of interest has been requested by several CerealsDB users. In response, we have created a webtool that can take two SNP IDs or a single SNP plus a specified distance in bps upstream or downstream of that SNP and the database will return a list of SNPs with the query SNP highlighted in red. Chromosomal location and physical positions are also indicated along with any annotations associated with the surrounding sequence. Many of the CerealsDB SNPs will have a putative annotation based on BLASTN alignments to the nr database. Clicking on the Axiom® probe names allows users to cross-reference this SNP with SNPs on other genotyping platforms that are hosted on CerealsDB.

#### Searching annotated Axiom® sequences

Located within the Axiom® SNP section of CerealsDB (accessed via the top menu bar) is a tool embedded in the right-hand vertical menu bar (called ‘Search axiom annotations’) that allows users to search for Axiom® probes that have been annotated by BLASTN alignment to the nr database. Users can enter the Axiom® code for a SNP, a GO term (e.g. GO:0048316, which is linked to seed development), a general search term such as ‘rust_resistance’ or a gene name, e.g. ‘spo11’. If the search term is found in the annotation table, the respective Axiom® SNPs are displayed along with the BLAST and GO terms (if available) and a weblink provided to the UniProt website (27) (a database of protein sequences and functional annotation) where more detailed information on the protein can be found.

Details on whether this SNP is available on one of the other genotyping platforms is also displayed using a colour-coded glyph that allows users to quickly scroll through hundreds of results and identify cross-platform SNPs (Figure 2). This is a useful functionality for plant breeders who frequently want to compare ‘legacy’ markers used in their breeding programmes. Clicking on the Axiom® code of a SNP provides more information on that SNP, such as alternative names, the Axiom® probe sequence, chromosomal location and any genetic mapping data, if available.

**Figure 3.**
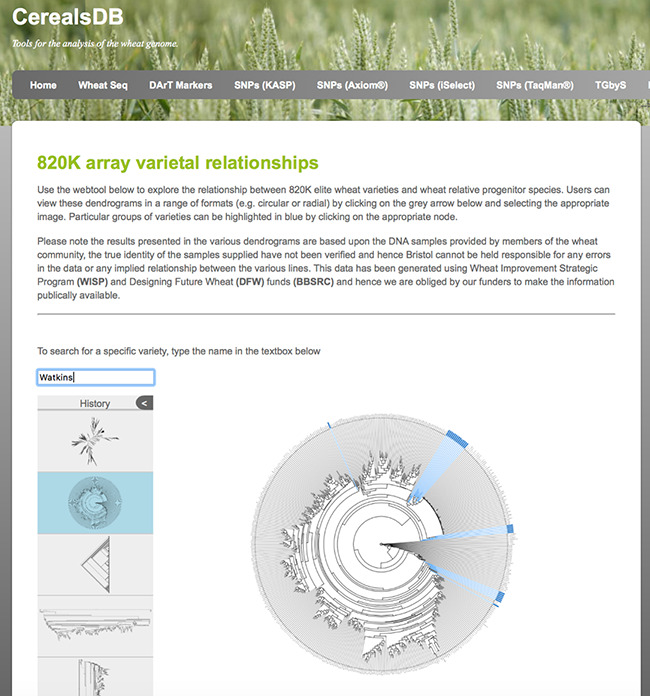
A circular dendrogram rendered using phylocanvas shows Watkins lines highlighted in blue. Users can view relationships between wheat varieties genotyped on both the 820K and 35K arrays and display the data in a number of different styles of dendrogram.

**Figure 4.**
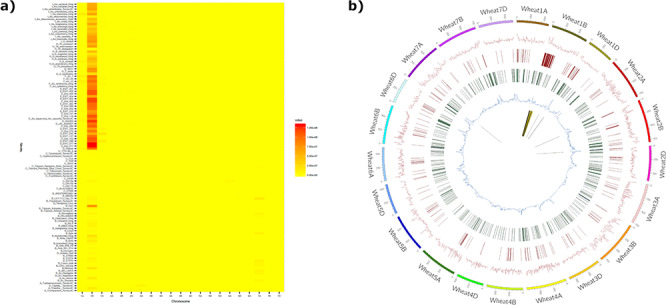
The introgression plotter generates a heatmap and a circos plot in this example for the variety Brompton. The heatmap (a) plots total introgression size for each comparison with relatives ordered vertically by relatedness to bread wheat and chromosomes ordered horizontally. Users can zoom in on the heatmap for more detail. The circos plot (b) has a number of tracks: track 1 (innermost track), putative introgressed/deleted regions; track 2, SNP density for each wheat chromosome; track 3, CNV gain; track 4, CNV loss; track 5, minor allele frequency. The outermost track represents wheat chromosomes (500 = 500 Mbp). The Brompton wheat variety is known to have the 1B/1RS translocation and this can clearly be seen in the circos plot.

#### Phylogenetic trees

Phylogenetic relationships between wheat varieties and wheat relative and progenitor species were investigated using Axiom® genotype calls. The SNPhylo (28) (version 20160204) pipeline was used to construct phylogenetic trees, separately for the 820K array and the 35K array datasets. These trees can be used to explore the genetic relationship between different accessions, for example, to understand the relationships between elite wheat varieties and wheat relative/progenitor species, revealing potentially interesting insights into the domestication of wheat and the development of modern ‘elite’ wheatvarieties.

It is possible to select one of three types of trees (circular, diagonal, hierarchical) and users can search for names of wheat varieties using the search field. Found names or text patterns will be immediately shown as blue tree nodes, with the names of the nodes given a blue background, which helps to locate varieties of interest in the tree. A subtree containing these selected varieties can be redrawn. Downloadable circular dendrograms for both datasets in high-resolution portable network graphic (png) format are also available and were generated using the R package ggtree (29). A screenshot of the 820K circular dendogram is displayed in Figure 3.

#### Introgression plotting tools

Two online webtools were created for viewing and interrogating putative introgressions/deletions. The first generates a heatmap of putative introgressions/deletions from all wheat relatives for each hexaploid wheat accession. The heat map is coloured according to the size of the putative introgression/deletions and allows users to determine which wheat relatives are possible donors. All heatmaps were pre-computed in R and exported as png images, which are embedded into the webpage. An associated interactive table details the total number and size of introgressions/deletions (in bps) detected for each relative comparison and allows users to sort and select a comparison to view in more detail.

The second tool generates circos plots to visualize putative introgressions/deletions from a single relative donor. This is combined with other parameters such as CNV, SNP density and genetic diversity measures for a straightforward comparison. An example of a heatmap and circos plot generated by the webtool is displayed in Figure 4, where the known 1B/1RS Rye introgression can clearly be seen in the circos plot when comparing the variety Brompton to wheat relative and progenitor species. The underlying introgression data for the plotter have also been used to confirm the presence of chromatin from the wheat wild relative *A. sharonensis* that was used to introgress stem rust resistance to bread wheat genomes (30). In Supplementary Figure 5, we can clearly see introgressed regions on chromosomes 1B and 5B for the ‘Pretorius’ wheat variety plotted as both heatmaps and circos plots.

## Conclusions

CerealsDB is a highly accessed site and an invaluable resource for both wheat researchers and breeders. All CerealsDB data are publicly available without restrictions and the database is continually updated with curated genotyping data. CerealsDB contains a range of tools for the visualization of wheat genomic datasets and facilitates integration with other databases via the web services that we have developed. Future plans include the extension of web services to offer further functionalities and a continuous increase in the number of genotyped varieties. In particular, we plan an expansion of the web services to include more genotyping and QTL data along with the streamlining of the site navigation to allow visitors to rapidly locate and retrieve the information that they are particularly interested in.

### Database architecture

CerealsDB version 3.0 uses a MySQL relational database management system database (version 14.14) running on a Linux server (Ubuntu OS version 18.04) and hosted using the Apache2 web server (version 2.4.7) with Perl, Python and PHP scripts used for all data retrieval and output. Genotyping data are also hosted in a MongoDB database to speed up retrieval of large genotyping datasets. The aforementioned software and architecture were used as they are platform independent and open source.

### Web services and availability

The CerealsDB website is freely available and can be accessed at http://www.cerealsdb.uk.net/cerealgenomics/CerealsDB/indexNEW.php. The site is free from IP restrictions and access is not password protected.

## List of abbreviations

API indicates application programming interface; BC, Bristol contig; BLAST, basic local alignment search tool; BS, Bristol SNP; CSS, cascading style sheets; DArT, diversity array technology; DFW, designing future wheat; EST, expressed sequence tag; GO, Gene Ontology; HTTP, hypertext transfer protocol; ID, identification; IWGSC, International Wheat Genome Sequencing Consortium; IWYP, International Wheat Yield Partnership; JSON, Javascript object notation; MAS, marker-assisted selection; NCBI, National Center for Biotechnology Information; NGS, next generation sequencing; nr, NCBI non-redundant database; OS, operating system; PHP, hypertext pre-processor; QTL, quantitative trait locus; RDMS, relational database management system; REST, representational state transfer; SNP, single nucleotide polymorphism; SNV, single nucleotide variant; SSD, single seed descent; TGbyS, targeted genotyping by sequencing; URGI, Unité de Recherche Génomique Info; URI, uniform resource identifier; URL, uniform resource locator; VCF, variant call format.

## Availability of data and materials

Datasets contained in CerealsDB are available on the site to download as Excel spreadsheets or VCF format. SNP data can be downloaded from the right-hand menu for each of the different genotyping platforms.

## Competing interests

The authors declare that they have no competing interests.

## Authors’ contributions

P.A.W., A.M.A., L.W., J.Z. and S.T. wrote the manuscript. P.A.W., G.L.A.B. and M.O.W. built CerealsDB version 3.0; P.A.W., S.T., X.B. and R.D. contributed to the web services; L.W. and S.G. provided the QTL data; A.M.A. and A.B. carried out the SNP validation experiments; D.S. and A.B. collated the varietal data; J.Z. predicted FATHMM scores; and K.J.E. and G.L.A.B. conceived of the study, participated in its design and coordination and helped to draft the manuscript. All authors have read and approved the final manuscript.

## Supplementary Material

CerealsDB_Supplementary_Data_revised_baaa060

## References

[1] Gupta P.K., Rustgi S. and Mir R.R. (2008) Array-based high-throughput DNA markers for crop improvement. Heredity, 101, 5–18. 18461083 10.1038/hdy.2008.35

[2] Wilkinson P.A., Winfield M.O., Barker G.L.A. et al. (2012) CerealsDB 2.0: an integrated resource for plant breeders and scientists. BMC Bioinformatics, 13, 219. 22943283 10.1186/1471-2105-13-219 PMC3447715

[3] Wilkinson P.A., Winfield M.O., Barker G.L.A. et al. (2016) CerealsDB 3.0: expansion of resources and data integration. BMC Bioinformatics, 17: 256. 27342803 10.1186/s12859-016-1139-x PMC4919907

[4] Winfield M., Allen A.M., Burridge A.J. et al. (2015) High density SNP genotyping array for hexaploid wheat and its secondary and tertiary gene pool. Plant Biotechnol. J., 14, 1195–1206. 26466852 10.1111/pbi.12485 PMC4950041

[5] Burridge A.J., Wilkinson P.A., Winfield M.O. et al. (2018) Conversion of array-based single nucleotide polymorphic markers for use in targeted genotyping by sequencing in hexaploid wheat (*Triticum aestivum*). Plant Biotechnol. J., 16, 867–876. 28913866 10.1111/pbi.12834 PMC5866950

[6] Bian X., Tyrrell S. and Davey R.P. (2017) The Grassroots life science data infrastructure. https://grassroots.tools.

[7] Winfield M.O., Wilkinson P.A., Allen A.M. et al. (2012) Targeted re-sequencing of the allohexaploid wheat exome. Plant Biotechnol. J., 10, 733–742. 22703335 10.1111/j.1467-7652.2012.00713.x

[8] Altschul S.F., Gish W., Miller W. et al. (1990) Basic local alignment search tool. J. Mol. Biol., 215, 403–410. 2231712 10.1016/S0022-2836(05)80360-2

[9] Binns D., Dimmer E., Huntley R. et al. (2009) QuickGO: a web-based tool for Gene Ontology searching. Bioinformatics, 25, 3045–3046. 19744993 10.1093/bioinformatics/btp536 PMC2773257

[10] Finn R.D., Clements J. and Eddy S.R. (2011) HMMER web server: interactive sequence similarity searching. Nucleic Acids Res., 39: W29–37. 21593126 10.1093/nar/gkr367 PMC3125773

[11] Suzek B.E., Wang Y., Huang H. et al. (2015) UniRef clusters: a comprehensive and scalable alternative for improving sequence similarity searches. Bioinformatics, 31, 926–932. 25398609 10.1093/bioinformatics/btu739 PMC4375400

[12] Sjolander K., Karplus K., Brown M. et al. (1996) Dirichlet mixtures: a method for improved detection of weak but significant protein sequence homology. CABIOS, 12, 327–345. 8902360 10.1093/bioinformatics/12.4.327

[13] Shihab H.A., Gough J., Cooper D.N. et al. (2013) Predicting the functional, molecular, and phenotypic consequences of amino acid substitutions using hidden Markov models. Hum. Mutat., 34, 57–65. 23033316 10.1002/humu.22225 PMC3558800

[14] Przewieslik-Allen S., Burridge A., Wilkinson P. et al. (2019) Developing a high-throughput SNP-based marker system to facilitate the introgression of traits from *Aegilops* species into bread wheat (*Triticum aestivum*). Front. Plant Sci., 9: 1993. 30733728 10.3389/fpls.2018.01993 PMC6354564

[15] Wingen L., West C., Leverington-Waite M. et al. (2017) Wheat landrace genome diversity. Genetics, 205, 1657–1676. 28213475 10.1534/genetics.116.194688 PMC5378120

[16] Allen A.M., Winfield M.O., Burridge A.J. et al. (2017) Characterisation of a Wheat Breeders’ Array suitable for high throughput SNP genotyping of global accessions of hexaploid bread wheat (*Triticum aestivum*). Plant Biotechnol. J., 15, 390–401. 27627182 10.1111/pbi.12635 PMC5316916

[17] Wu Y., Bhat P.R., Close T.J. et al. (2008) Efficient and accurate construction of genetic linkage maps from the minimum spanning tree of a graph. PLoS Genet., 4, e1000212. 18846212 10.1371/journal.pgen.1000212 PMC2556103

[18] Broman K.W., Wu H., Sen S. et al. (2003) R/QTL: QTL mapping in experimental crosses. Bioinformatics, 19, 889–890. 12724300 10.1093/bioinformatics/btg112

[19] Shrestha R., Arnaud E., Mauleon R. et al. (2010) Multifunctional crop trait ontology for breeders’ data: field book, annotation, data discovery and semantic enrichment of the literature. AoB Plants, 2010, plq008. 22476066 10.1093/aobpla/plq008 PMC3000699

[20] https://github.com/phylocanvas/phylocanvas

[21] Horler R.S.P., Turner A.S., Fretter P. et al. (2018) SeedStor: a germplasm information management system and public database. Plant Cell Physiol., 59(1). 10.1093/pcp/pcx195 PMC5914401 29228298

[22] Bolser D., Staines D.M., Pritchard E., Kersey P. (2016) Ensembl Plants: Integrating Tools for Visualizing, Mining, and Analyzing Plant Genomics Data In: Edwards D (ed). Methods Mol Biol., 1374, 115–140. 26519403 10.1007/978-1-4939-3167-5_6

[23] Wolfe D., Dudek S., Ritchie M.D. et al. (2013) Visualizing genomic information across chromosomes with PhenoGram. BioData Min., 6, 18. 24131735 10.1186/1756-0381-6-18 PMC4015356

[24] Xiuling T., Weie W., Li X. et al. (2017) Molecular mapping of reduced plant height gene Rht24 in bread wheat. Front. Plant Sci., 8, 1379. 28848582 10.3389/fpls.2017.01379 PMC5550838

[25] Würschum T., Langer S.M., Longin C.F.H. et al. (2017) A modern green revolution gene for reduced height in wheat. Plant J., 92, 892–903. 28949040 10.1111/tpj.13726

[26] Griffiths S., Simmonds J., Leverington M. et al. (2012) Meta-QTL analysis of the genetic control of crop height in elite European winter wheat germplasm. Mol. Breed., 29, 159–171. 10.1007/s00122-009-1046-x 19430758

[27] The UniProt Consortium (2015) UniProt: a hub for protein information. Nucleic Acids Res., 43, D204–D212. 25348405 10.1093/nar/gku989 PMC4384041

[28] Lee T.H., Guo H., Wang X. et al. (2014) SNPhylo: a pipeline to construct a phylogenetic tree from huge SNP data. BMC Genomics, 15: 162. 24571581 10.1186/1471-2164-15-162 PMC3945939

[29] Yu G., Smith D.K., Zhu H. et al. (2017) ggtree: an R package for visualization and annotation of phylogenetic trees with their covariates and other associated data. Methods Ecol. Evol., 8, 28–36.

[30] Millet E., Steffenson B.J., Prins R. et al. (2017) Genome targeted introgression of resistance to African stem rust from *Aegilops sharonensis* into bread wheat. Plant Genome., 10(3). 10.3835/plantgenome2017.07.0061 29293809

