# Supplementary material for: CerealsDB—new tools for the analysis of the wheat genome: update 2020
Source: Database (Oxford). 2020 Aug 4;2020:baaa060. doi: 10.1093/database/baaa060 (PMC7402920; doi:10.1093/database/baaa060)

**Supplementary Data**

**Supplementary Table 1: Axiom SNP statistics for each chromosome.**

| **Chromosome** | **Number of SNP-containing contigs** | **Average no. Of SNPs per Contig** | **Minimum no. of SNPs per Contig** | **Max no. Of SNPs per Contig** |
| --- | --- | --- | --- | --- |
| 1A | 5404 | 4.82143 | 1 | 97 |
| 1B | 5869 | 4.41915 | 1 | 98 |
| 1D | 5621 | 4.93097 | 1 | 118 |
| 2A | 7397 | 4.67027 | 1 | 157 |
| 2B | 8086 | 4.50866 | 1 | 90 |
| 2D | 8216 | 4.39301 | 1 | 99 |
| 3A | 5808 | 4.26222 | 1 | 81 |
| 3B | 6676 | 5.0358 | 1 | 72 |
| 3D | 6074 | 4.14554 | 1 | 61 |
| 4A | 6463 | 4.42442 | 1 | 78 |
| 4B | 6155 | 4.17742 | 1 | 74 |
| 4D | 6105 | 4.49402 | 1 | 71 |
| 5A | 5340 | 4.57846 | 1 | 70 |
| 5B | 7959 | 4.60787 | 1 | 118 |
| 5D | 6374 | 5.43991 | 1 | 86 |
| 6A | 5000 | 4.7412 | 1 | 83 |
| 6B | 4775 | 4.38094 | 1 | 127 |
| 6D | 4886 | 5.44269 | 1 | 149 |
| 7A | 5308 | 4.47155 | 1 | 117 |
| 7B | 5017 | 4.43432 | 1 | 79 |
| 7D | 5897 | 5.33966 | 1 | 92 |

**Supplementary Table 2: Most common annotations of contigs containing CerealsDB SNPs.**

| **Annotation** | **Number of annotated SNP sequences** |
| --- | --- |
| Disease resistance gene | 10535 |
| Cytochrome p450 | 3681 |
| Subtilisin-like protease | 1058 |
| Pentatricopeptide repeat-containing protein | 951 |
| Secologanin synthase | 905 |
| B3 domain-containing protein | 872 |
| Wall-associated receptor kinase 3 | 858 |
| Protein srg1 | 717 |
| O-methyltransferase zrp4 | 703 |
| Peptide transporter ptr2 | 622 |
| Peroxidase 2 | 573 |
| Putative receptor protein kinase zmpk1 | 547 |
| Myb-related protein | 495 |
| 1-phosphatidylinositol-3-phosphate 5-kinase fab1 | 489 |
| Aspartic proteinase nepenthesin-2 | 483 |
| Auxin-induced protein 5ng4 | 461 |
| Aspartic proteinase nepenthesin-1 | 429 |
| Putative receptor-like protein kinase | 414 |
| Peroxidase 12 | 388 |
| Protein far1-related sequence 5 | 380 |
| Putative o-methyltransferase 2 | 376 |
| Glycosyltransferase | 367 |
| Putative s-acyltransferase | 352 |
| Tom1-like protein 2 | 342 |
| Protein transparent testa | 340 |

**Supplementary Table 3: Proportion of 820K SNPs used in the SNPhylo analysis**

| Chromosome | Number SNPs | Number SNPs used in SNPhylo analysis | % SNPs used |
| --- | --- | --- | --- |
| 1A | 24043 | 2513 | 10.45% |
| 1B | 29075 | 4479 | 15.40% |
| 1D | 41485 | 3747 | 9.03% |
| 2A | 30415 | 3009 | 9.89% |
| 2B | 36154 | 4482 | 12.40% |
| 2D | 51938 | 4724 | 9.10% |
| 3A | 27472 | 1997 | 7.27% |
| 3B | 33631 | 2815 | 8.37% |
| 3D | 48220 | 2622 | 5.44% |
| 4A | 26762 | 1751 | 6.54% |
| 4B | 28052 | 1306 | 4.66% |
| 4D | 36061 | 922 | 2.56% |
| 5A | 31466 | 2256 | 7.17% |
| 5B | 35253 | 3534 | 10.02% |
| 5D | 49661 | 2908 | 5.86% |
| 6A | 22627 | 2034 | 8.99% |
| 6B | 28686 | 3133 | 10.92% |
| 6D | 36727 | 2238 | 6.09% |
| 7A | 27800 | 2581 | 9.28% |
| 7B | 31036 | 2829 | 9.12% |
| 7D | 46582 | 2673 | 5.74% |

**Supplementary Table 4: Proportion of 35K SNPs used in the SNPhylo analysis**

| Chromosome | Number SNPs | Number SNPs used in SNPhylo analysis | % SNPs used |
| --- | --- | --- | --- |
| 1A | 1258 | 700 | 55.64% |
| 1B | 1806 | 850 | 47.07% |
| 1D | 1741 | 740 | 42.50% |
| 2A | 1403 | 846 | 60.30% |
| 2B | 1729 | 1094 | 63.27% |
| 2D | 1886 | 1082 | 57.37% |
| 3A | 1255 | 665 | 52.99% |
| 3B | 1429 | 797 | 55.77% |
| 3D | 1571 | 695 | 44.24% |
| 4A | 907 | 527 | 58.10% |
| 4B | 980 | 522 | 53.27% |
| 4D | 772 | 297 | 38.47% |
| 5A | 1337 | 810 | 60.58% |
| 5B | 1458 | 952 | 65.29% |
| 5D | 1404 | 770 | 54.84% |
| 6A | 954 | 646 | 67.71% |
| 6B | 1400 | 912 | 65.14% |
| 6D | 1020 | 570 | 55.88% |
| 7A | 1351 | 771 | 57.07% |
| 7B | 1364 | 710 | 52.05% |
| 7D | 1383 | 671 | 48.52% |

**Supplementary Figure 1:**

Unique visits to CerealsDB May 2012 - Feb 2020

**Supplementary Figure 2:** Global distribution of varieties by country, showing the majority of wheat varieties contained in CerealsDB have come from donors in the UK.


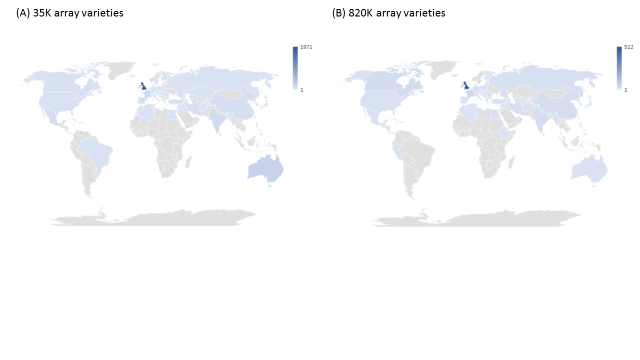


**Supplementary Figure 3:**

A schematic showing how CerealsDB interacts with a MongoDb database hosting genotyping information at the John Innes Centre and uses the Grassroots API base at the Earlham institute to access the genotyping data.

**
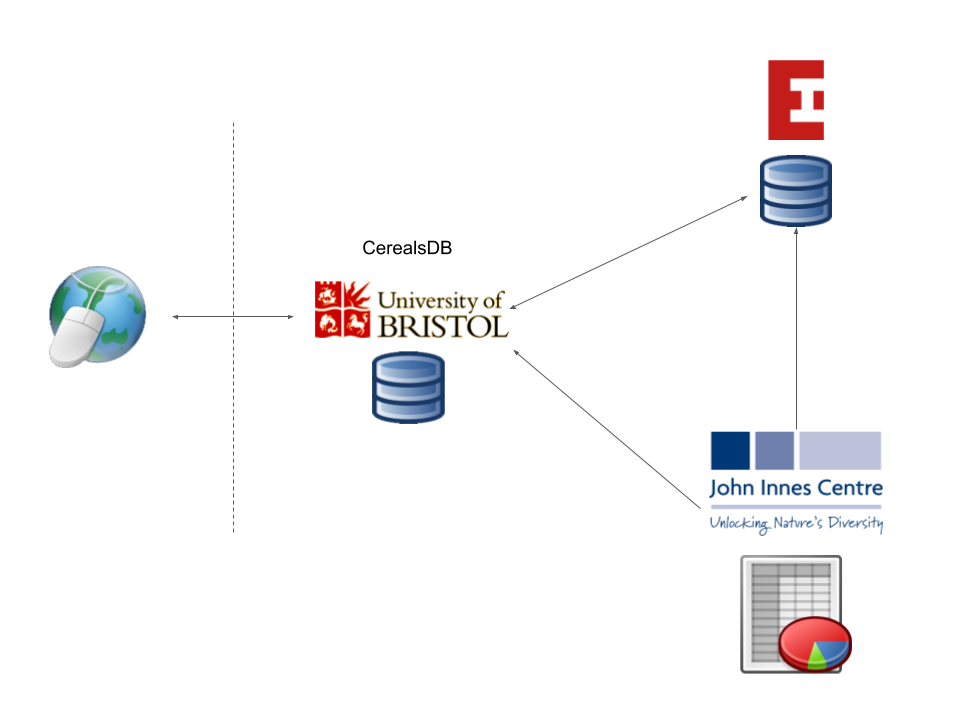
**

**Supplementary Figure 4:** Alignment of six plant height QTL confidence intervals along the IWGSC whole genome assembly RefSeq-v1.0 of chromosome 6A. The ruler to the left shows the base pair position. Chromosomes or QTL confidence interval regions on linkage groups are depicted as vertical light blue bars. Markers on chromosomes or linkage groups are given as horizontal lines, with the marker name on the right. The bar to the left, directly next to the ruler and labelled as RefSeq-v1-6A, depicts the whole genome assembly of chromosome 6A. Only a few significant markers that helped to identify the *Rht24* locus are given. The three markers in purple are the markers suggested by Würschum et al. (2017) for marker assisted selection for this locus. All other vertical bars are the QTL confidence intervals for plant height on 6A, this is the information easily available from the QTL database. The abbreviation of the bi-parental population used for QTL discovery is given on top of the bar. All markers are positioned according to their physical position on the whole genome assembly. Markers nearest to the QTL peak are given in blue. Identical markers on adjacent chromosomes are linked by vertical lines.


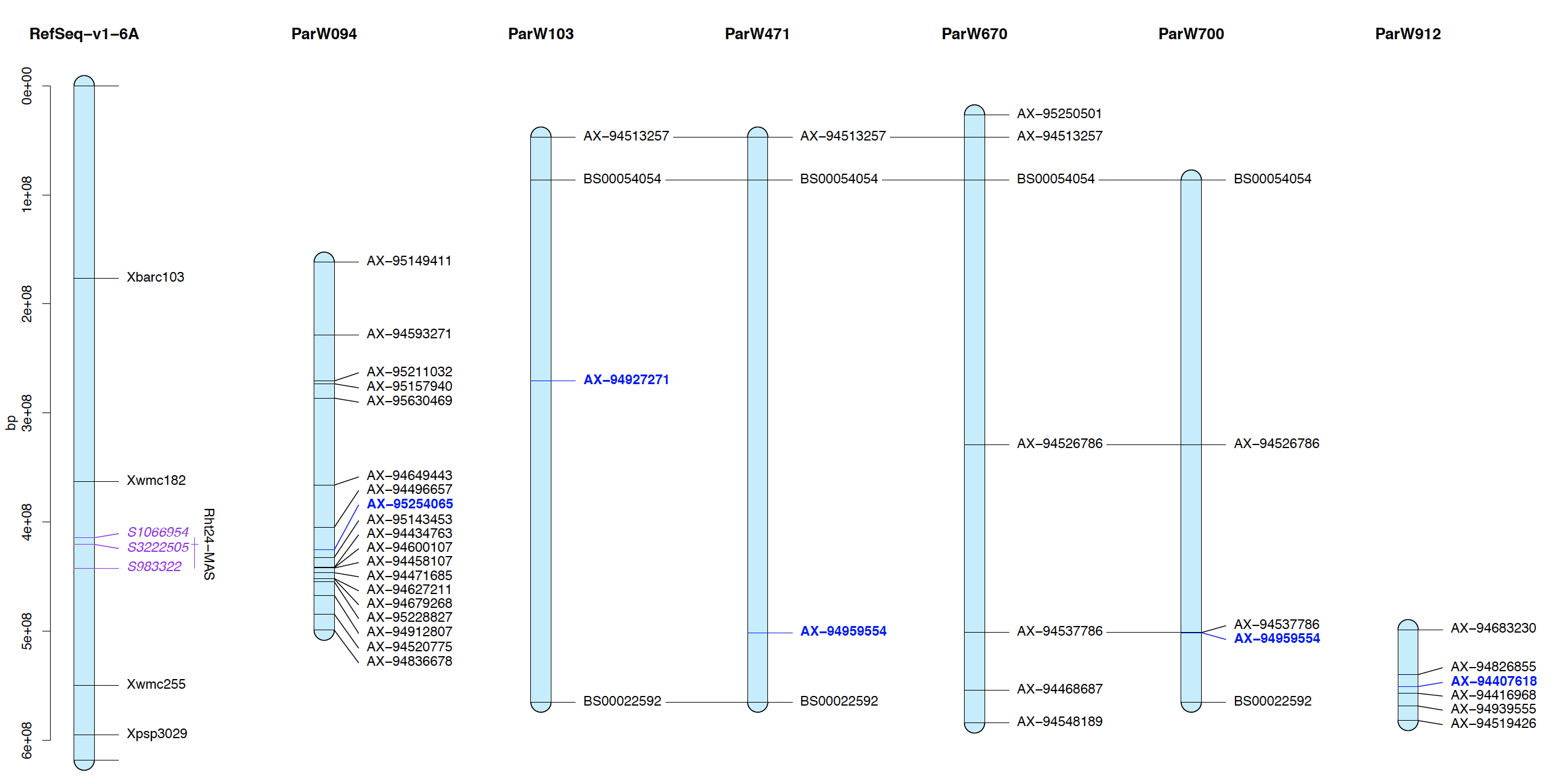


**Supplementary Figure 5:** A heat map and circus plot showing an *Aegilops* introgression detected in the ‘Pretorius’ wheat varieties. An introgression on chromosome 1B is shown in Figure 4a and an introgression on chromosome 5B is shown in Figure 4b.


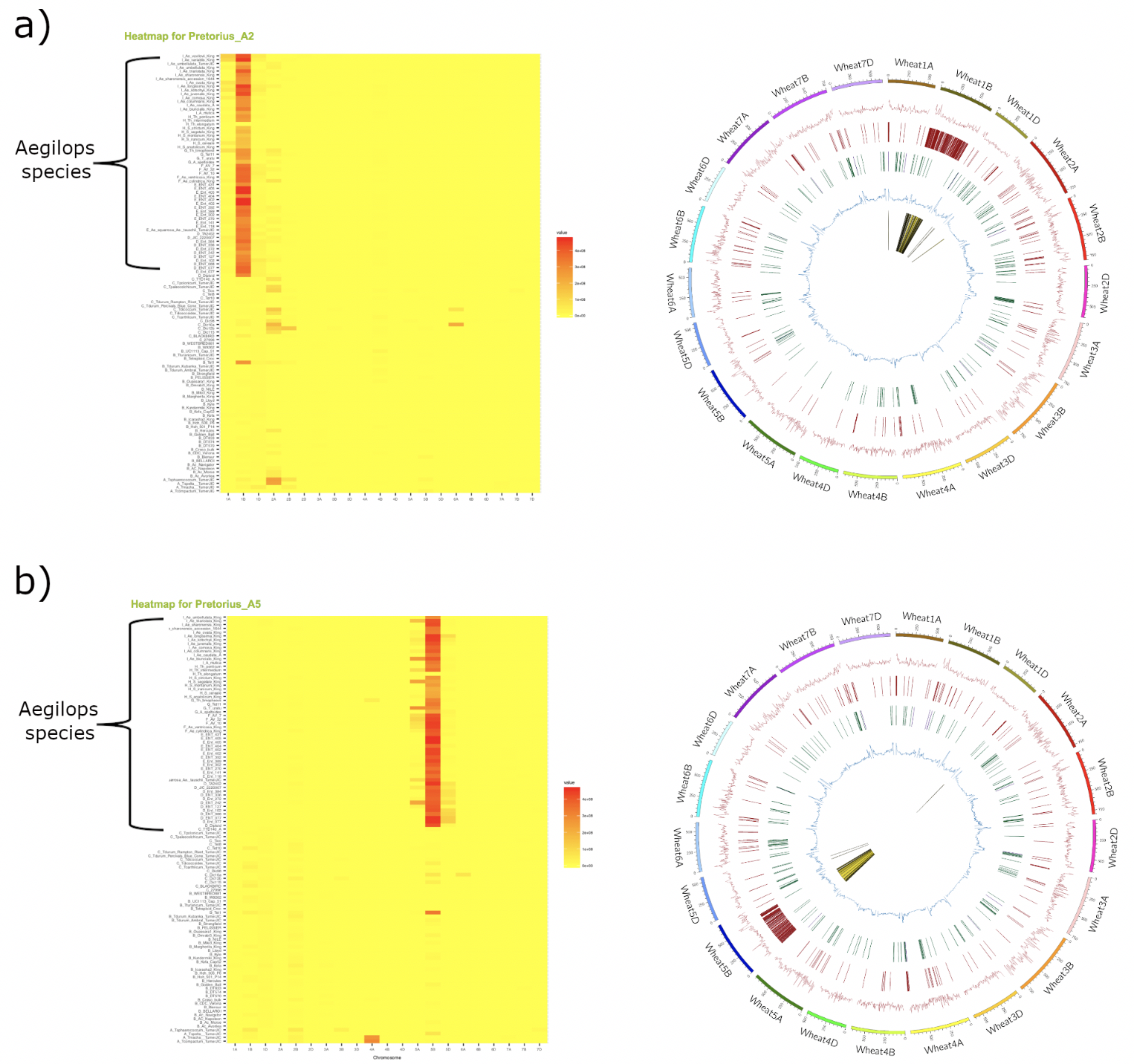

Supplement: CerealsDB_Supplementary_Data_revised_baaa060 [file cerealsdb_supplementary_data_revised_baaa060.docx]
